# The Immuno-Modulatory Effects of Inhibitor of Apoptosis Protein Antagonists in Cancer Immunotherapy

**DOI:** 10.3390/cells9010207

**Published:** 2020-01-14

**Authors:** Jessica Michie, Conor J. Kearney, Edwin D. Hawkins, John Silke, Jane Oliaro

**Affiliations:** 1 Peter MacCallum Cancer Centre, Melbourne, VI 3000, Australia; 2 Sir Peter MacCallum Department of Oncology, The University of Melbourne, VI 3010, Australia; 3 The Walter and Eliza Hall Institute of Medical Research, VI 3010, Australia; silke@wehi.edu.au; 4 Department of Medical Biology, University of Melbourne, Parkville, VI 3010, Australia; 5 Department of Immunology, Monash University, Melbourne, VI 3004, Australia

**Keywords:** smac-mimetics, TNF, cancer immunotherapy, checkpoint blockade, CAR T cells

## Abstract

One of the hallmarks of cancer cells is their ability to evade cell death via apoptosis. The inhibitor of apoptosis proteins (IAPs) are a family of proteins that act to promote cell survival. For this reason, upregulation of IAPs is associated with a number of cancer types as a mechanism of resistance to cell death and chemotherapy. As such, IAPs are considered a promising therapeutic target for cancer treatment, based on the role of IAPs in resistance to apoptosis, tumour progression and poor patient prognosis. The mitochondrial protein smac (second mitochondrial activator of caspases), is an endogenous inhibitor of IAPs, and several small molecule mimetics of smac (smac-mimetics) have been developed in order to antagonise IAPs in cancer cells and restore sensitivity to apoptotic stimuli. However, recent studies have revealed that smac-mimetics have broader effects than was first attributed. It is now understood that they are key regulators of innate immune signalling and have wide reaching immuno-modulatory properties. As such, they are ideal candidates for immunotherapy combinations. Pre-clinically, successful combination therapies incorporating smac-mimetics and oncolytic viruses, as with chimeric antigen receptor (CAR) T cell therapy, have been reported, and clinical trials incorporating smac-mimetics and immune checkpoint blockade are ongoing. Here, the potential of IAP antagonism to enhance immunotherapy strategies for the treatment of cancer will be discussed.

## 1. Inhibitor of Apoptosis Proteins

The capacity to evade apoptosis, a form of physiological cell death that relies on the activation of a family of cysteine proteases known as caspases [1], is a common trait of malignantly transformed cells [2]. During apoptotic cell death, endogenous second mitochondrial activator of caspases/Direct IAP-Binding Protein With Low PI (smac/DIABLO), is released from the mitochondrial inter-membrane space where it binds to, and inhibits, the three major inhibitor of apoptosis proteins; cellular IAP 1 (cIAP1, *BIRC2*) and 2 (cIAP2, *BIRC3*) and X-linked IAP (XIAP, *BIRC4*) [3,4]. The inhibitor of apoptosis (IAP) proteins are a family of endogenous proteins that function as key regulators of caspase activity, and are defined by the presence of at least one Baculoviral IAP Repeat (BIR) domain. These approximately 70-residue zinc-binding domains enable their interaction with, and suppression of, caspases, and therefore facilitate the inhibition of apoptosis [5]. Only XIAP is a potent direct inhibitor of caspases, however, the physiological significance of this activity is unclear, because cells from patients with XIAP mutations [6] and murine XIAP knockout mice, are not more sensitive to apoptosis than wild type cells [7]. Importantly, IAPs also contain a RING finger E3 ligase domain at the C-terminus [8,9], enabling these proteins to participate in diverse cellular processes, including signal transduction events that promote inflammation, cell cycle progression and migration. Notably, IAPs are critical regulators of both canonical and alternative (non-canonical) nuclear factor kappa light-chain enhancer of activated B cells (NF-κB) signalling, downstream of various members of the Tumour Necrosis Factor Receptors Superfamily (TNFRSF).

### 1.1. Inhibitor of Apoptosis Proteins in NF-κB Signalling

**Figure 1:**
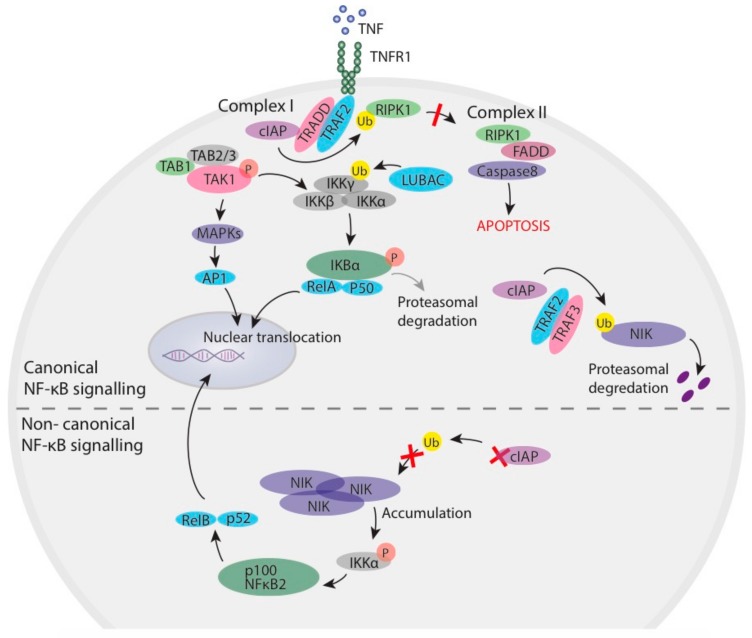
The Inhibitor of Apoptosis Proteins (IAPs) are critical regulators of both canonical and non-canonical NF-κB signalling. During canonical NF-κB signalling, the ubiquitylation of Complex I components by cIAPs results in the nuclear translocation and activation of pro-survival canonical NF-κB and limits the formation of pro-apoptotic Complex II. cIAPs also target NIK for proteasomal degradation preventing the activation of non-canonical NF-κB. Loss of IAPs results in the formation of Complex II and activates caspase-mediated apoptosis, and results in the accumulation of NIK, which causes downstream non-canonical NF-κB activation.

IAPs are required for the activation of the canonical NF-κB pathway downstream of several receptors [10,11]. One of the best studied is downstream of TNF Receptor 1 (TNFR1) (Figure 1). In this pathway, TNFR1 ligation by TNF results in the formation of a complex comprising RIPK1, TRADD, and TRAF2 (Complex I), where TRAF2 is the primary factor required for the recruitment of IAPs [12,13,14]. IAPs ubiquitylate several components within this complex, although the best studied is RIPK1 [15,16,17,18]. The downstream signalling pathway consists of the trimeric canonical IκB kinase (IKK) complex, composed of IKKα and IKKβ subunits, as well as the regulatory subunit IKKγ (also known as NF-κB essential modulator (NEMO)). IAP-mediated ubiquitylation of Complex I mediates the recruitment of the linear ubiquitin chain assembly complex (LUBAC) [19], which is comprised of HOIL-1L, HOIP and Sharpin [20]. LUBAC generates M1 linked ubiquitin chains on Complex I components such as RIPK1 and IKKγ [21], which stabilizes Complex I and allows full activation of the IKK complex (consisting of IKK1, IKK2 and IKKγ/NEMO) and a TAK1 containing complex. IKK2 phosphorylates IκBα, resulting in its proteasomal degradation and the release of the p50 and p65/RelA NF-κB heterodimer, which allows their translocation to the nucleus [22,23], while TAK1 activation leads to activation of the MAPK pathway. This results in the induction of pro-survival and inflammatory transcriptional programs [24].

IAP-mediated ubiquitylation of RIPK1 in Complex I also limits RIPK1 association with FADD and caspase 8 to form the ripoptosome (Complex II) [25]. Together MAPK, IKK activation and IAP ubiquitylation therefore suppress TNF induced apoptosis. As a result, antagonism, or the absence of, IAPs results in signalling through TNFR1 that activates caspase-mediated apoptosis, rather than the induction of NF-κB pro-survival signalling [26,27,28]. IAPs also inhibit cell death induced by the TNFSF death ligands, FasL and TRAIL, as well as chemotherapy agents [28,29,30,31]. In these cases, it is likely that preventing RIPK1 from generating a FADD caspase-8 activating complex is the primary mechanism of inhibition, while NF-κB activation plays a less significant role.

The cellular IAPs (cIAPs) also have a critical role in the regulation of NF-κB-inducing kinase (NIK) through K-48 ubiquitylation; a process that leads to its continuous degradation and therefore low-level expression [26,27]. Therefore, the loss or inhibition of IAPs leads to NIK stabilization and accumulation, phosphorylation and activation of IKKα, phosphorylation and processing of p100 (NFκB2) and nuclear translocation of the p52 and RelB NF-κB heterodimer; resulting in activation of the so-called non-canonical NF-κB pathway.

### 1.2. IAPs and Smac-Mimetics in Cancer

IAPs are amplified in approximately 3% of cancers [32], and several IAPs can act as proto-oncogenes as a result of genetic alterations in various cancer types [33]. As IAPs promote cell survival in response to TNFSF ligands and chemotherapy, their upregulation renders cancer cells resistant to the cytotoxic effects of these agents, and is associated with failure of treatment and poor prognosis in multiple cancer types [24,25,34]. For example, XIAP expression has been implicated in cisplatin resistance in ovarian cancer [35], correlated with disease severity in AML [36], and is a prognostic indicator in renal cell carcinoma [37]. Following the discovery of a potent antagonist of XIAP [38], small molecule mimetics of smac/DIABLO (smac-mimetics; SMs) were developed to overcome the survival benefits of IAP upregulation in cancer cells, through their antagonism of IAP activity and promotion of tumour cell death [39,40,41,42]. SMs promote IAP degradation by binding to the BIR3 domain of cIAPs, which allows the RING to dimerize, and in turn, activate the E3 ubiquitin ligase function of the cIAPs [26,27,43]. As they are not bound to a natural substrate such as NIK, this results in their own ubiquitylation and degradation [44,45]. Although SMs also bind to the BIR3 of XIAP, very few of them promote degradation of XIAP, although they nevertheless can antagonise normal XIAP function [11,46,47].

A number of SMs have been developed over the past decade with differing chemistries, including monovalent and bivalent varieties, which both mimic the N-terminus of SMAC and subsequently promote RIPK1-caspase8 complex formation and apoptosis. However, bivalent SMs are typically more cytotoxic than the structurally related monovalent IAP antagonists. Biochemical analyses of various monovalent and bivalent SMs have revealed that bivalency promotes cIAP1 degradation more effectively than monovalent IAP antagonists, and monovalent SMs are also less effective at depleting TRAF2-associated cIAP1, and thus inhibiting canonical NF-κB signalling [48]. SMs not only degrade IAPs to promote tumor cell death, but also induce the production of TNF in a subset of tumor cells, which can act in an autocrine or paracrine manner to cause tumor cell death [24,26,27,49,50]. In this manner, single agent efficacy of SMs has been reported, particularly for the treatment of certain leukaemias [51,52,53]. However, these monotherapy studies have shown that, for the most part, antagonism of IAPs alone does not result in immediate cell death; rather, SM-induced assembly of the ripoptosome can prime cells for death—a process which requires an additional RIPK1 activating signal [1,30,31], which lowers the threshold for TNF-dependent apoptotic cell death. The importance of the ripoptosome in SM-induced cell death was highlighted by a recent study demonstrating resistance to SM therapy by chronic lymphocytic leukaemia cells as the result of an inability to form the ripoptosome complex [54]. SMs do not usually sensitize non-transformed cells to TNF-induced death, as is evident from the good safety profile of these drugs, but how healthy cells are protected from this effect is not well understood.

Several SMs have now entered clinical trials, including monovalent LCL161 and Debio 1143 (AT- 406) and one of the most clinically advanced SMs—the bivalent and pan-IAP antagonist birinapant [39]. Despite promising pre-clinical efficacy, this has largely failed to translate into positive outcomes in clinical trials, with several reports of the poor efficacy of SMs when used as single agents [32,55]. However, due to their favourable safety profile, attention is now being directed to the incorporation of SMs in combination therapies and as immuno-modulatory agents for the treatment of cancer.

### 1.3. The Immuno-Modulatory Properties of Smac-Mimetics in Cancer

Our understanding of the physiological roles of IAPs has undergone a transformation since they were originally identified as a potential target for cancer therapy. Their functions in regulating activation of canonical NF-κB, inhibiting cell death and inhibiting activation of the non-canonical NF-κB pathway all play a role in regulating inflammatory and immune responses in normal development and disease [56,57,58,59,60]. Both the IAPs and NF-κB signalling play a critical role in the normal functioning of the immune system, including in B cell development [57,61], in the regulation of myeloid cells [62] and in cytotoxic lymphocyte activity (see later sections). For this reason, SMs have been reported to exhibit broad immuno-modulatory effects on both the innate and adaptive immune systems.

The immuno-modulatory effects observed following SM therapy, along with the ubiquitous inflammatory properties of TNF, have led to a number of studies reporting the effects of SMs on the immune system in cancer. TNF-dependent anti-tumour activity mediated by bacillus Calmette-Guérin (BCG)-stimulated neutrophils following SM treatment has been reported [63]. In murine models of human ovarian carcinoma or murine sarcoma, SM administration was associated with a rapid inflammatory burst of TNF and interferon-gamma (IFNγ), due to the reversion of tumour-associated macrophages from a pro-tumoural M2 phenotype to a pro-inflammatory MI-like phenotype [64]. The SM compound BV6, can sensitize resistant tumour cells to killing by cytokine-induced killer cells [65] and synergise with interferon-alpha (IFNα) to induce apoptosis in acute myeloid leukeamia cells [66]. Synergy between IFNγ and various SMs, including BV6 and the clinical compound birinapant, has been reported to induce apoptosis in lung cancer cells [67,68]. The pre-clinical efficacy of LCL161, a SM which potently induces apoptosis in some multiple myeloma cell lines with over-expression of XIAP has been reported [69], and can also sensitise osteosarcoma cells to TNF-mediated apoptosis [70].

The administration of SMs also stimulates myeloma cells to secrete soluble factors that induce tumour cell phagocytosis by macrophages; demonstrating the immune-modulatory properties of SMs [32]. SM-mediated activation mimicking CD40 engagement was directly responsible for inducing an inflammatory response in macrophages and dendritic cells in vitro, which was further potentiated in vivo by the presence of SM-sensitive tumour cells. Two weeks of LCL161 treatment was curative in 15% of tumour bearing mice, an anti-myeloma response that was attributed to activation of tumour cell type I interferon signalling, resulting in myeloid activation and tumour cell phagocytosis, rather than direct cell killing [32].

As SMs become more clinically advanced, there is increasing data on the immunomodulatory effects of SMs in patients. In some instances, clinical data corroborates the pre-clinical findings, suggesting that SMs have wide-reaching effects on immunity. A phase 1 study of LCL161 against solid tumours of lung, skin, colon, pancreas and other origins (NCT01098838) determined pharmacodynamic activity with a favourable safety profile up to 1800 mg weekly, where cytokine release syndrome was the dose-limiting toxicity in 60% of patients [71]. Phase 2 evaluation of LCL161 against relapsed refractory multiple myeloma (NCT01955434) demonstrated some efficacy, with a partial response achieved in a number of patients. Immuno-modulatory properties of LCL161 were observed in patients following treatment, where a type I interferon response was required for activity, induction of inflammatory cytokines in the bone marrow plasma of patients after three weeks of LCL161 treatment was observed, and there was evidence of increased macrophage-mediated phagocytosis [32]. However, other SM clinical studies show less profound immunomodulation. In Phase 1 (NCT00993239) and 2 (NCT01098838) clinical trials of birinapant, no signficant differences in serum levels of TNF, interleukin-6 (IL-6) or IL-8 were observed regardless of dose [55,72], and only modest effects on immune cell populations were observed [55].

While SMs are likely to be effective as a monotherapy only where tumours release autocrine TNF following the degradation of IAPs, innate immune stimuli capable of inducing a clinically safe cytokine storm in the context of the tumour microenvironment may result in significant bystander killing in the presence of SMs [73]. As a result, there are now significant research efforts being directed towards the use of IAP antagonists as part of combination strategies that incorporate various immunotherapy approaches.

## 2. IAP Antagonists and Immunotherapy Approaches

Immunotherapy has had some remarkable successes in the clinic, however the benefit is limited to only a subset of patients. For example, checkpoint blockade therapy, such as α-PD-1 treatment, results in a partial or complete clinical response rate in only ~20% of patients with solid tumours [74]. The application of chimeric antigen receptor (CAR) T cell therapy has also been limited to haematological malignancies, largely due to poor CAR T cell infiltration and the hostile tumour microenvironment associated with solid tumours. Several properties of SMs, including their immuno-modulatory effects on a range of immune subsets, may overcome these challenges and enhance the efficacy of immunotherapies when used in combination. SM therapy can also synergise with innate immune stimuli such as oncolytic viruses, poly(I:C) or CpG, to promote anti-tumour immunity in mouse models [50]. The potential of SMs to promote the anti-tumour activity of CD8+ cytotoxic T lymphocytes (CTLs) is particularly promising, as these are the key immune cells required for the clinical efficacy of both adoptive cell therapies and checkpoint blockade immunotherapies.

### 2.1. Role of Smac-Mimetics in T Cell-Specific Immunotherapies

CTLs possess remarkable anti-tumour cytolytic capacity, and therefore are key targets for enhancing anti-tumour immunity. Activation of naïve T cells occurs following the interaction between a peptide antigen presented in the context of major histocompatibility complex and the T cell receptor (TCR). T cell activation also requires co-stimulation through the interaction of the T cell receptor, CD28, and the membrane bound glycoproteins, CD80 or CD86, expressed on professional antigen presenting cells, such as dendritic cells [75]. In CD4+ T cells, ligation of CD28 promotes T cell survival through the induction of NF-κB-dependent expression of anti-apoptotic BCL-XL [76]. Appropriate T cell activation occurs following both activating signals, which results in T cell proliferation, cytokine production and antigen-specific killing of target cells.

Upon activation, T cells express several co-stimulatory TNFRSF members, including TNFR2, OX40 and 4-1BB, which all activate NF-κB2 in a cIAP dependent manner [77]. In this way, cIAPs can function as negative regulators of T cell co-stimulation [58,78]. Activating non-canonical NF-κB signalling following SM treatment is therefore likely to affect all T cell subsets. Indeed, inhibiting cIAPs in human and murine CD4+ and CD8+ T cells delivers a co-stimulatory signal that enhances activation in the presence of TCR stimulation [79]. Overall, this results in an enhancement of CD8+ and CD4+ T cell proliferation and functions such as cytokine production [78], and may therefore augment immunity by stimulating the adaptive immune response in vivo.

Moreover, it has been shown that the bivalent SM, APG-1387, positively regulates T cells in vitro, reducing regulatory T (Treg) cell differentiation and downregulating PD-1 expression on CD4+ T cells [80]. More recently, the molecular and functional imprinting capacity of SM treatment on T cells at the transcriptional and proteomic level was demonstrated, where non-canonical NF-κB signalling was shown to skew non-differentiated T cells into a more Th2-like, and less Th17-like, differentiation state [81]. While the direct effects of SMs on T cells are still being uncovered, it is clear that they can modulate T cell activation and differentiation, which will have important consequences when combined with immunotherapy approaches that target and promote the anti-tumour activity of T cells. It is not yet known if SMs will elicit co-stimulatory effects on T cells that are activated though a synthetic chimeric antigen receptor, which might have clinical applications in the context of adoptive CAR T cell therapies for cancer.

The pro-inflammatory properties of smac-induced cell death play an important role in T cell mediated anti-tumour immunity; highlighted in a study where delivery of the cytosolic form of smac resulted in immunogenic tumour cell death and triggered an anti-tumour immune response leading to decreased tumour growth in two melanoma models, and total tumour regression in a mastocytoma model [82]. In this instance, dendritic cells were activated upon uptake of dying tumour cells, resulting in increased functional CTL numbers and decreased Treg numbers. Administration of SMs also improved the outcome of both prophylactic and therapeutic anti-tumour vaccines in vivo, with the SM, LBW-242, reported to synergise with a vaccine against B16 melanoma, delaying tumour growth and prolonging survival [79].

The TNF-mediated apoptotic pathway is a potent mechanism of inducing tumour cell death [83], and IAP antagonists rapidly sensitise tumour cells to killing by CTL-derived TNF. More recently, the potential of agents, such as birinapant, that lower the tumour TNF cytotoxicity threshold and augment responses to immunotherapy has been demonstrated [84]. Indeed, T cell killing in vitro is enhanced following the addition of birinapant—an effect that was TNF-dependent [85]. In support of these findings, a synergistic enhancement of T cell effector function due to birinapant and α-PD-1 co-administration was reported; an effect attributed to enhanced TNF secretion [32]. Intracranial glioblastoma cells are also sensitive to the effects of IAP antagonists (either LCL161 or birinapant) in combination with exogenous TNF or TRAIL [86]. SMs synergised with immune checkpoint inhibitors to promote tumour immunity against glioblastoma, an effect that was dependent on both CD8+ T cells and TNF. SM and immune checkpoint blockade combination therapy was associated with increased expansion of CD8+ T cells, a decrease in Treg numbers and increased sensitivity of the tumours to TNF-induced cell death mediated through the RIPK1-Fadd-Caspase8 complex. Based on the pre-clinical success of SM and checkpoint blockade combination strategies, phase I/2 clinical trials are on-going to assess the toxicity and efficacy of the combination of these treatment modalities, including birinapant, APG-1387, LCL161 and Debio1143 (Table 1).

**Figure 2:**
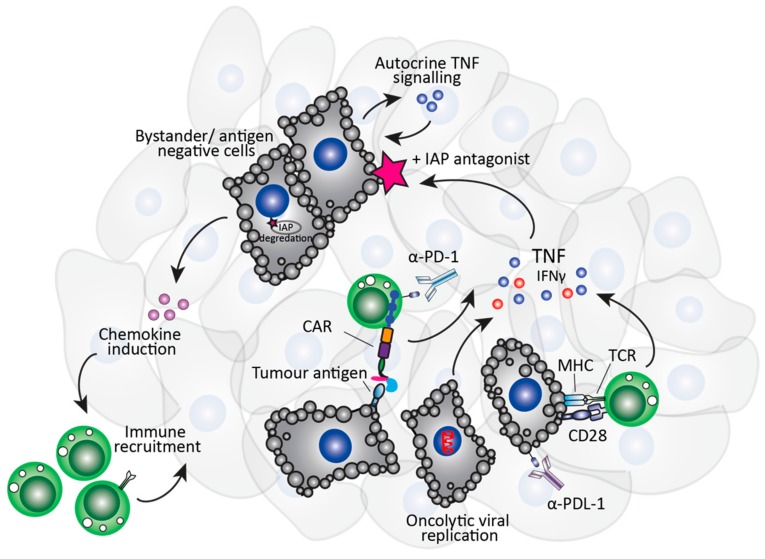
Bystander killing mediated by tumour cell IAP antagonism. TNF released following checkpoint blockade, CAR T cells, or from tumour cells following oncolytic virus-induced death can cause death of surrounding “bystander” tumour cells, due to IAP degradation.

Birinapant also significantly enhanced CAR T cell therapy, resulting in prolonged survival and tumour regression in tumour bearing mice, and significantly enhanced tumour cell death in a patient-derived tumoroid model, compared to CAR T cell or birinapant monotherapy [87]. Importantly, the ability of SMs to senstitise antigen negative cells to TNF-mediated apoptosis, an effect known as bystander killing (Figure 2), may be an effective strategy to avoid antigen negative relapse following CAR T cell therapy [88]. Harnessing tumour-antigen specific CAR T cells as a vehicle to deliver TNF specifically to the tumour in combination with birinapant may have a significant therapeutic advantage. CAR T cells secrete inflammatory cytokines only following tumour antigen recognition through the CAR, which avoids the potential detrimental effect of systemic TNF secretion. α-PD-1 therapy increases CAR T cell-derived TNF [89], suggesting that the combination of CAR T cell therapy, SM treatment and checkpoint blockade may be a potent treatment strategy that could be rapidly translated into the clinic.

SMs can also mediate the induction of systemic cytokines such as TNF, and chemokines such as RANTES and CXCL1 [90], which results in enhanced immune cell recruitment in vivo. In combination with adoptive cell therapy, SMs may therefore enhance the trafficking of immune cells into the tumour microenvironment. TNF produced by immune cells contributes to SM efficacy, particularly against tumours that do not secrete TNF following degradation of IAPs. As a result of this, SM are more efficacious in vivo when combined with inflammatory adjuvants that promote TNF production [50,90,91]. Overall, SMs can elicit a pro-inflammatory cell death in cancer cells that engages an adaptive anti-tumour immune response, and may result in an increase in tumour-infiltrating lymphocytes, cytokine secretion and enhance inflammation in an otherwise immunologically “cold” tumour.

The SM, Debio 1143, also has radiosensitization properties in non-small cell lung cancer [92] and head and neck squamous cell carcinoma; an effect that has been attributed to enhanced TNF sensitivity [93]. However, the immune-modulatory properties of SMs in this context have only recently been explored. Synergistic anti-tumour effects between Debio 1143 and ablative radiotherapy have been reported, where addition of the SM not only induced a tumour-specific CD8+ T cell and cytokine response, but was able to overcome the recruitment of immunosuppressive host cells into the tumour microenvironment [94]—which typically occurs following radiotherapy. As a result of this pre-clinical efficacy, a phase I clinical trial combining re-irradiation therapy and birinapant for the treatment of head and neck squamous cell carcinoma is ongoing (NCT03803774).

### 2.2. Role of Smac-Mimetics in NK Cell-Specific Immunotherapies

While the success of CAR T cell therapy and checkpoint blockade has highlighted the use of T cell-centric immunotherapies for the treatment of cancer, there is potential for natural killer (NK) cell-specific immunotherapies to provide an alternative or overcome relapse or resistance to these therapies [95,96]. Like T cells, IAPs also regulate aspects of NK cell biology, however their role has not been extensively studied.

There have been several reports that IAP antagonists potentiate Hodgkin lymphoma susceptibility towards NK cell-mediated killing [97,98], and a TNF-dependent increase in the effector function of NK cells in vitro following the administration of birinapant has been reported [85]. Similarly, BV6 improves NK cell-mediated killing of rhabdomyosarcoma cells by sensitising them to NK cell-mediated killing through TNF, whilst simultaneously enhancing the cytotoxic potential of the NK cells through increasing NK cell activation [99]. Furthermore, the administration of AG-1387 resulted in a five-fold increase in NK cell tumour infiltration, as well as inducing tumour cell apoptosis directly in a hepatocellular carcinoma xenograph model [80]. More recently, the cytotoxic activity of NK cells against hepatocellular carcinoma was shown to be enhanced following AG-1387 administration [100]. Based on this favourable pre-clinical efficacy, and mounting evidence for the role of the PD-1/PDL-1 inhibitory axis on NK cell cytotoxic activity [101,102,103], a Phase 1/2 clinical trial with AG-1387 in combination with pembrolizumab (α-PD-1), is ongoing (Table 1). IAP activity, particularly XIAP, is also implicated in NKT cell development [104], and SM treatment has been reported to enhance cytokine production from mouse and human NKT cells and promote the anti-tumour activity of NKT cells in a B16F10 lung metastasis model [105], providing excellent rationale to combine SMs with immunotherapy approaches.

Whether NK cell-based immunotherapies will become commonplace in clinical practice is still unknown, but some clinical success has been reported following the adoptive transfer of the NK92 cell line or NK cells engineered to express a chimeric antigen receptor against a tumour associated antigen [106,107]. The role of NK cells in anti-tumour immunity should not be underestimated, and given the potent cytotoxic capacity of NK cells to kill tumour cells, the potential to enhance their effector function with SMs is a promising approach. Indeed, emerging literature suggests that tumour cells may be senstitised to NK cell-mediated and TNF-dependent killing through administration of a SM, and potentially overcome resistance to T cell-directed immunotherapies [96].

### 2.3. Role of Smac-Mimetics in Oncolytic Virus Immunotherapy

Oncolytic virus immunotherapy is a treatment modality that uses genetically modified viruses that selectively replicate within, and directly lyse, tumour cells. This treatment has emerged as a promising tool to treat solid tumours, due to their remarkable capacity to selectively replicate in cancer cells without affecting non-malignant cells [108]. Indeed, clinical trials have reported clinical benefit in patients following oncolytic viral immunotherapy [109,110].

Following oncolytic cell death, tumour cells release tumour-associated neo-antigens, viral pathogen associated molecular patterns (PAMPs) and danger associated molecular patterns (DAMPs), which serve to induce an innate and adaptive immune response against cancer cells. Combined with the release of cytokines from tumour cells, this promotes the activation of CD4+ and CD8+ T cells, which traffic to sites of established tumour cell growth and mediate anti-tumour immunity, releasing more inflammatory cytokines into the tumour microenvironment [111]. The use of oncolytic viral immunotherapy to modulate the tumour microenvironment and release inflammatory cytokines provides a strong rationale for combination with other immunotherapies, such as checkpoint blockade [112,113] and/or SMs, to improve therapeutic responses.

Indeed, SMs have shown impressive and broad activity when combined with oncolytic viruses [91], likely due to the increase in local TNF production as a result of oncolytic cell death. The combination of SMs and oncolytic virus therapy, both of which have significant immunomodulatory properties, can synergise to promote anti-tumour T cell responses due to apoptosis of SM-treated cells triggered by TNF secreted during viral infection [114]. Treatment with the oncolytic virus, M1, through the release of associated PAMPs, results in an increase in inflammatory cytokine secretion via the host innate immune system. SMs also sensitise tumour cells to inflammatory cytokines induced by M1, enhancing a bystander killing effect mediated by IL-8, IL1A and TRAIL [115].

The generation of oncolytic viruses that express TNF resulted in an enhanced anti-tumour effect when combined with LCL161, due to an increase in TNF-mediated bystander killing and collapse of tumour-vasculature [116]. It has also been reported that SMs themselves may have tumour-vasculature targeting properties, through the sensitisation of endothelial cells to TNF-mediated death [117]. Taken together, these studies highlight the multi-purpose role of SMs in promoting anti-tumour immunity and the potential for combination therapies that harness the favourable effects of SMs on the immune system.

## 3. Conclusions

While IAPs were originally characterised as modulators of cell death through their interaction with caspases, it is now clear that they have broad immuno-modulatory properties, affecting both the innate and adaptive immune system. As such, IAP antagonists are poised to enter the clinic as a promising approach to overcome some of the challenges associated with conventional immunotherapies. One of the key immuno-modulatory effects of SMs is their ability to potentiate TNF-mediated bystander killing of tumour cells.

Combination therapies that enhance the efficacy of SMs are those that typically increase the local concentration of inflammatory cytokines, which can then be harnessed and potentiated by SMs via their capacity to sensitise tumour cells to TNF-mediated apoptosis. Therefore, therapies specifically designed to enhance the local secretion of inflammatory cytokines in the tumour microenvironment, such as CAR T cell or oncolytic virus therapy, have resulted in a synergistic effect with SMs and significantly enhanced tumour cell death. It may be possible to create triple combination therapies based on this approach, by incorporating an immune checkpoint blockade–a therapy which has been shown to enhance T cell secretion of inflammatory cytokines–to further enhance this synergistic effect. As many of these agents with potential for synergy with SMs are already in clinical trials, we can expect a rapid translation of such combination therapies in to the clinic.

## Figures and Tables

**Table 1:** Summary of smac-mimetics in clinical trials incorporating an immunotherapy approach.

Identifier	Smac- mimetic	Combination Therapy	Malignancy	Phase
**NCT03270176**	Debio 1143 (AT- 406)	Avelumab (α-PDL-1)	Advances solid tumours and advanced/metastatic non-small cell lung carcinoma	1
**NCT02587962**	Birinapant (TL32711)	Pembrolizumab (α-PD-1)	Various solid tumours	1/2
**NCT03111992**	LCL161	PDR001 (α-PD-1) CJM112 (anti-IL-17a)	Multiple myeloma	1
**NCT02890069**	LCL161	PDR001 (α-PD-1) Everolimus (mToR inhibitor) Panobinostat (HDAC inhibitor)	Colorectal cancer, non-small cell lung carcinoma, triple negative breast cancer, renal cell carcinoma	1
**NCT03166631**	BI891065	B I754091 (α-PD-1)	Advanced and/or metastatic malignancies	1
**NCT03697304**	BI891065	BI 754111 (anti-LAG-3) BI 754091 (α-PD-1)	Advanced and/or metastatic solid tumours (pre-treated)	2
**NCT03871959**	Debio 1143	Pembrolizumab (α-PD-1)	Non-MSI-high advanced or metastatic pancreatic ductal adenocarcinoma or colorectal cancer	1
**NCT03386526**	APG-1387	Pembrolizumab (α-PD-1)	Advanced solid tumours or haematological malignancies	1/2
